# Coupled Heterogeneity to Dimeric Site-Specific Binding by the POU-Family Transcription Factor OCT2

**DOI:** 10.1021/acs.jpcb.4c07071

**Published:** 2025-02-17

**Authors:** J. Ross Terrell, Gregory M. K. Poon

**Affiliations:** Department of Chemistry, Georgia State University, Atlanta, Georgia 30303, United States

## Abstract

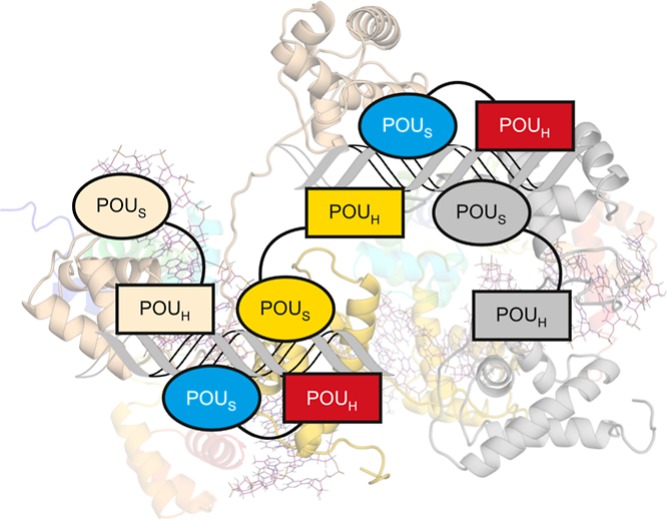

POU-family transcription factors regulate metazoan gene expression via a bipartite DNA-binding domain consisting of two covalently linked helix-turn-helix subdomains, POU_S_ and POU_H_. POU factors bind as dimers to DNA half-sites to form complexes with a variable quaternary structure. To enhance the knowledge of the physical chemistry of dimeric POU/DNA recognition, we carried out a crystallographic and titration analysis of the cooperative homodimer formed by the POU factor OCT2 and an optimized palindromic DNA site known as MORE. The data evidence strong heterogeneity in the binding and formation of secondary complexes in site-specific DNA recognition by OCT2 at thermodynamic equilibrium. These secondary complexes are strictly contingent to the site-specific complex, detectable at subsaturating OCT2 concentrations, and cooperate with nonspecific binding to guide the affinity of the site-specific complex. Modulation with salt and poly[d(I-C)] unmasks the compensation between nonspecific DNA depleting unbound OCT2 on the one hand while driving specific binding by intermolecular transfer of OCT2 via secondary complexes on the other. Molecular dynamics simulations extend a mechanism, previously proposed for POU monomers, in which the two subdomains dynamically cross-link DNA strands to form supramolecular dimeric POU/DNA complexes at equilibrium.

## Introduction

Quaternary structure is a molecular rationale for the low number of structural families that comprise human transcription factors.^1^ Members of the bZIP,^2^ nuclear receptor,^3^ Sox families,^4^ for example, heterodimerize with other members of their respective families to form functionally distinct complexes in addition to partnerships with transcription factors from structurally unrelated families. Human transcription factors typically exhibit a modular domain architecture in which their DNA-binding domains (DBDs) are flanked by disordered regions.^5^ Modularity facilitates combinatorial diversity by balancing the specification of quaternary structure between protein–protein complementarity and programmable DNA sequence.^6^

Beyond modularity in the domain structure, the POU (Pit-Oct-Unc) family of transcription factors presents modularity within their DBDs, known as the POU domain. The POU domain combines two helix-turn-helix motifs, a tetra-helical POU-specific subdomain (POU_S_) and a triple-helical homeodomain (POU_H_)^7^ via a linker. This bipartite arrangement enables POU factors to select among a spectrum of related DNA sequences depending on the length, spacing, and orientation of POU_S_- and POU_H_-binding motifs and form a wide variety of complexes facultatively as dimers and monomers.^8−11^ Some dimeric configurations are stabilized by favorable contacts between the two subdomains, which are stably folded structures on their own,^12,13^ a canonical example being the OCT1 complex with the so-called PORE sequence.^14^ However, intersubdomain contacts are not required for site-specific or high-affinity DNA binding,^15^ and many POU factors bind single octamer motifs stably as monomers. The length and composition of the linker interact with the DNA sequence to determine the structure and affinity of site-specific complexes,^16^ though it is typically unresolved in crystallographic and solution NMR studies. In certain POU members, such as OCT4, the linker is not fully disordered but contains a short α-helix that mediates interactions with cofactor proteins.^17^

Biophysically, POU domains are classic model systems of DNA-facilitated target search by multimeric transcription factors. Simulations^18^ and NMR spectroscopic studies^19,20^ have established mechanisms by which the two POU_X_ (X = S or H) subdomains in a POU monomer can slide, hop, and especially exchange between DNA strands, a process known as intersegmental transfer. Beyond this, little is known about the physicochemical driving forces of POU/DNA recognition, particularly in a dimeric configuration. To this end, we carried out a crystallographic and titration analysis of dimeric POU/DNA binding by human POU factor OCT2 (POU2F2). OCT2 belongs to Class II of the POU family, which includes OCT1, another archetypal POU factor, and PLA1. We solved a cocrystal structure of OCT2 bound to an optimized palindromic sequence, 5′-ATGCATATGCAT-3′ known as MORE (“More palindromic Oct factor Recognition Element”),^21^ which is also a functionally important POU-binding motif in chromatin.^22^ OCT2^23^ as well as other POU factors dimerize cooperatively on dyadic sequences such as MORE, with several (OCT1,^14^ Oct6,^24^ and Brn2^25^) having been crystallized with it. MORE was therefore well suited for probing the physical chemistry of dimeric OCT2/DNA binding. The cocrystal structure revealed nonequivalence in the subunits of the OCT2 homodimer, consistent with topologic heterogeneity of the POU_X_ subunits. In solution, cooperative binding to MORE was markedly accompanied by secondary supramolecular species that were not nonspecific but thermodynamically coupled to the site-specific complex. Taken together, these results reveal intrinsic heterogeneity in dimeric POU/DNA interactions and suggest a role in target site localization.

## Materials and Methods

### Nucleic Acids

Synthetic oligonucleotides were purchased from Integrated DNA Technologies (Midland, IA). Lyophilized oligonucleotides were dissolved at 100 μM in 30 mM HEPES, pH 7.5, containing 100 mM potassium acetate and annealed (with complementary sequences when required) by heating to 90 °C followed by passive cooling to room temperature. Biosynthetic poly d(IC) (sodium salt) was obtained from Roche, dissolved at 10 OD (1 OD = 6.9 × 10^–3^ M cm^–1^ bp at 251 nm)^26^ in water and used without further purification.

### Expression and Purification of the OCT2 DBD

A double-stranded fragment encoding the DBD (residues 195 to 357) of human OCT2 was cloned into the NdeI and BamHI sites of the pET15b vector (Novagen). To overexpress the N-terminally His_6_-tagged protein construct, transformed BL21(DE3)pLysS *E. coli* were cultured in LB medium to 0.6 OD at 37 °C, induced with 0.5 mM IPTG, and maintained at 18 °C overnight. Harvested cells were resuspended in Buffer N (10 mM HEPES, pH 7.4, containing 0.3 M NaCl, and 5 mM β-mercaptoethanol), lysed by sonication, and cleared by centrifugation at 40,000*g*. Cleared lysates were loaded onto Ni-NTA resin (HisTrap HP, Cytiva), washed with Buffer N, and eluted in Buffer N containing 0.5 M imidazole. The eluate was treated with 0.2 U/L thrombin (MP Biomedicals) overnight to cleave the His_6_ tag, diluted to 0.2 M NaCl, and loaded onto Sepharose SP (HiTrap SP HP, Cytiva). After washing with Buffer H (10 mM HEPES, pH 7.4, containing 0.15 M NaCl), elution was carried out over a NaCl gradient under the control of an FPLC instrument (NGC; Bio-Rad). Target fractions were concentrated using a 10,000 MWCO centrifugal device (Millipore) and further purified on a HiLoad 16/600 Superdex 75 (Cytiva) column in Buffer H. TCEP was added to 0.5 mM. Protein concentration was measured by UV absorption at 280 nm based on an extinction coefficient of 12,490 M^–1^ cm^–1^.

### X-ray Crystallography

Purified protein was concentrated in 10 k MWCO centrifugal filters and mixed with MORE DNA (5′-TCCTCATGCATATGCATGAGGA-3′) at a 2:1 stoichiometric ratio in Buffer H to yield a complex concentration of 200 μM. Crystallization conditions were screened using the JCSGplus (Molecular Dimensions) and Index HT (Hampton Research) crystal screens in 96-well plates with a sitting-drop configuration. Condition H12 (150 mM KBr, 30% PEG MME 2000) from the Index HT screen produced well-formed crystals. Condition H12 was replicated in hanging-drop plates and the crystals grown in a well containing 150 mM KBr, 27.5% PEG MME 2000 harvested directly and flash-frozen for data collection without additional cryoprotection. Diffraction data were collected at Brookhaven National Laboratory (Upton, NY) on the 17-ID-1 AMX Beamline at 0.920105 Å wavelength. The data were autoprocessed using XDS and processed data cut using the data reduction suite in CCP4i2. Phasing was carried out in Phenix through molecular replacement using maximum-likelihood search procedures in PHASER-MR (search model derived from PDB: 1E3O) followed by cycles of refinement and modeling in phenix.refine and Coot, respectively. Collection and refinement statistics are given in Table S1 (Supporting Information).

### Electrophoretic Mobility Shift

A DNA fragment harboring a single copy of the MORE site was cloned into the EcoRI and HindIII sites of pUC19 and PCR-amplified by using M13-based primers. The sequence of the 0.2-kbp DNA fragment is given in Figure S1 (Supporting Information). Gel-purified DNA probe (10 nM) was incubated overnight at 25 °C with graded concentrations of purified OCT2 DBD in 20 μL of PBS buffer. After Ficoll was added to 0.5% as the loading dye, the sample was resolved in a 4–20% gradient polyacrylamide gel running at 20 V/cm. Following electrophoresis, the gel was stained with GelRed (Biotium) and digitized with a LAS 4000 mini (Cytiva) imager under 520 nm excitation.

### Fluorescence Polarization Titrations

5′-hexachlorofluorescein (HEX)-conjugated DNA probe encoding the MORE sequence (5′-TCCTCATGCATATGCATGAGGA-3′; 5 nM) was incubated with graded concentrations of the OCT2 DBD to equilibrium (overnight at 25 °C) in 10 mM Bis-Tris, pH 6.5 (to suppress cysteine disulfide formation), 0.01% bovine serum albumin (BSA), and 0.15 to 0.35 M NaCl. Steady-state fluorescence anisotropies *r* were measured at 595 nm in a Molecular Dynamics Paradigm microplate reader with 530 nm excitation. Anisotropies were reported as means ± SD of three or more experiments and related to probe (P) and OCT2 (O) concentrations as follows
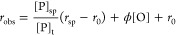
1where the subscripts “sp” and “0” refer to the specifically bound (saturable) and unbound probe, and the subscript “t” refers to the total probe concentration. Excess binding was modeled as a linear coefficient ϕ of the free titrant (OCT2) concentration.^27^

### Model-Dependent Analysis of Quantitative Titrations

Site-specific OCT2 binding to the MORE probe [P]_sp_ was fitted to the Hill equation
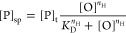
2where *K*_D_ is the macroscopic equilibrium dissociation constant and *n*_H_ = 2 on account of the highly cooperative 2:1 binding to the MORE sequence (vide infra). In addition, the equations of state for P and O were incorporated into the model to explicitly handle the total concentration of the ionized OCT2 as the independent variable
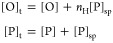
3

The concentrations of the various species were solved by numerical root finding of the nonlinear system of equations consisting of eqs 2 and 3 using the function c05qbc from the NAG C (Numerical Algorithms Group, Oxford, UK) library and fitted to the anisotropy data through eq 1 using Origin software (Northampton, MA).

### Molecular Dynamics Simulations

Explicit-solvent simulations were performed with the Amber14SB/bsc1 force field^28^ in the GROMACS 2024.x environment. The initial coordinates for the OCT2 DBD were constructed by modeling the amino acid sequence onto the cocrystal OCT2/DNA structure using I-TASSER.^29^ Optimized parm94-compatible charges for inosine were used as reported.^30^ Each system was set up in dodecahedral boxes at least 1.0 nm wider than the longest dimension of the solute, solvated with TIP3P water, and neutralized with Na^+^ and Cl^–^ to 0.15 M. Electrostatic interactions were handled by particle-mesh Ewald summation with a 1 nm distance cutoff. Where required, simulations were carried out at 298 K and 1 bar. A time step of 2 fs was used, and H-bonds were constrained using LINCS. After the structures were energy-minimized by steepest descent, the *NVT* ensemble was equilibrated at 298 K (v-rescale) for 1 ns to thermalize the system, followed by another 1 ns of equilibration of the *NPT* ensemble at 1 bar (C-rescale) and 298 K. The final *NPT* ensemble was simulated without restraints for 200 ns, recording coordinates every 1 ps. Convergence of the trajectories were checked by RMSD from the energy-minimized structures, after correcting periodic boundary effects. Triplicate production runs were carried out using different random seeds in the velocity distribution. Postsimulation analyses were performed using facilities provided by GROMACS. Rotational correlation times were computed by integration of a second-order Legendre expansion of the autocorrelation function, constructed using half the number of frames (typically 5000).

## Results

### Co-Crystal Structure of the OCT2/MORE Complex

**Figure 1:**
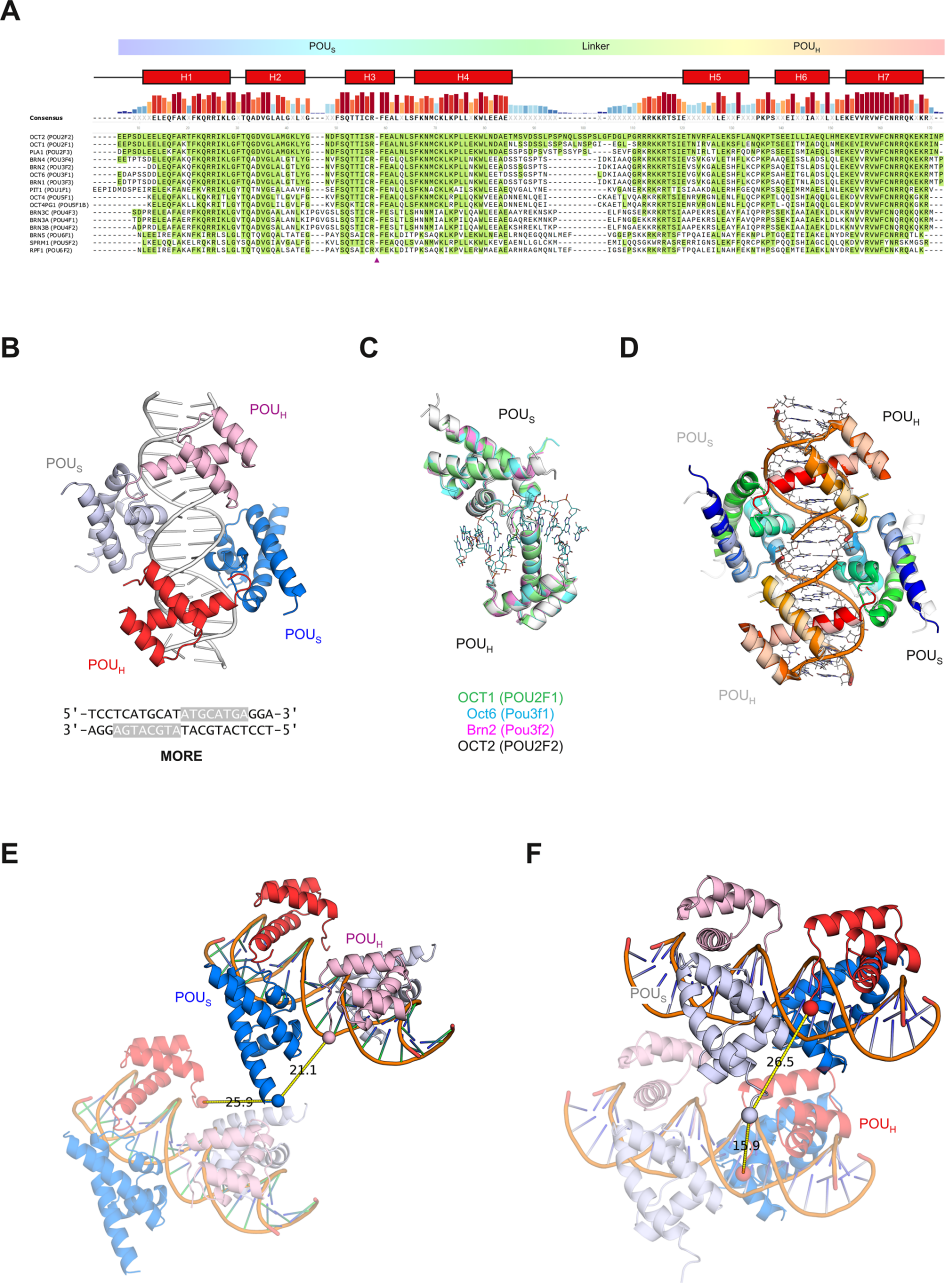
The OCT2/MORE cocrystal structure. (A) Multiple sequence alignment of human POU-family factors captured by a blast search of the OCT2 DBD against the UniProt database. The purple triangle marks the position in which RPF1 (POU6F2) encodes a 36-residue loop without homology among the other POU members and was omitted for space. (B) Overall structure of the OCT2/DNA complex and sequence of the optimized palindromic MORE site in the asymmetric unit. Each pair of POU_X_ (**X** = S or H) subdomains is colored without assumption of their covalent linkage. (C) Structural alignment of resolved protein elements in the OCT2 structure with three other POU factors cocrystallized with MORE: OCT1 (PDB: 1E3O), Oct6 (2XSD), and Brn2 (7XRC). (D) Superposition of the OCT2/MORE asymmetric unit (gray) with two adjacent crystallographic units of the OCT1/MORE structure (colored). (E,F) Distance (in Å) between the C-terminus of a POU_S_ subdomain and the N-termini of the two closest POU_H_ subdomains. The resolved termini are shown as spheres. Only the nearest pair of asymmetric units (one rendered semitransparent) in the lattice are shown for clarity.

The OCT2/MORE complex (PDB ID: 9DZM) crystallized as a homodimer on the MORE sequence, as expected for this palindrome (Figure 1A). Despite the dyad symmetry in MORE, however, the asymmetric unit consisted of the OCT2 homodimer bound with the full sequence containing the two inverted octamers, in contrast with the monomer/single octamer seen in asymmetric units of the MORE-bound cocrystals of OCT1,^14^ Oct6,^24^ and Brn2.^25^ The resolved portions of the two OCT2 subunits aligned albeit incompletely with each other and are in good general agreement with their counterparts in the OCT1, Oct6, and Brn2 structures (Figure 1B). The DNA termini were also nonequivalent, with one terminus in Watson–Crick base pairing but the other frayed. Nevertheless, the trajectory of the DNA in the OCT2/DNA structure fitted the 2-fold crystallographic symmetry in the other MORE-bound structures (Figure 1C).

To address the crystallographic asymmetry, we hypothesized that heterogeneity was introduced through disordered elements not resolved in the structure. To this end, we examined crystal contacts near the expected locations of the linkers, i.e., the C-terminal side of POU_S_ and N-terminal side of POU_H_. For one POU_S_ subdomain, the closest POU_H_ subdomain was the one in the asymmetric unit, although it was also only ∼5 Å farther from an equivalent POU_H_ subdomain in an adjacent unit (Figure 1D). In contrast, the other POU_S_ subdomain was located >10 Å closer to a POU_H_ subdomain in an adjacent asymmetric unit (Figure 1E). A model based on nearest neighbors would assign one pair of POU_X_ subdomains axially along the DNA within the same asymmetric unit, as in other MORE-bound structures, while placing the other subunit in a cross-link across adjacent asymmetric units. As OCT2 carries the longest linker among POU factors^22^ (Figure 1A), other combinations not limited by linker length are also possible. The evidence therefore suggested that the OCT2 adopted mixed linker topologies in the OCT2/MORE crystal lattice.

### Secondary Complexes Are Coupled to the Site-Specific OCT2/DNA Complex

**Figure 2:**
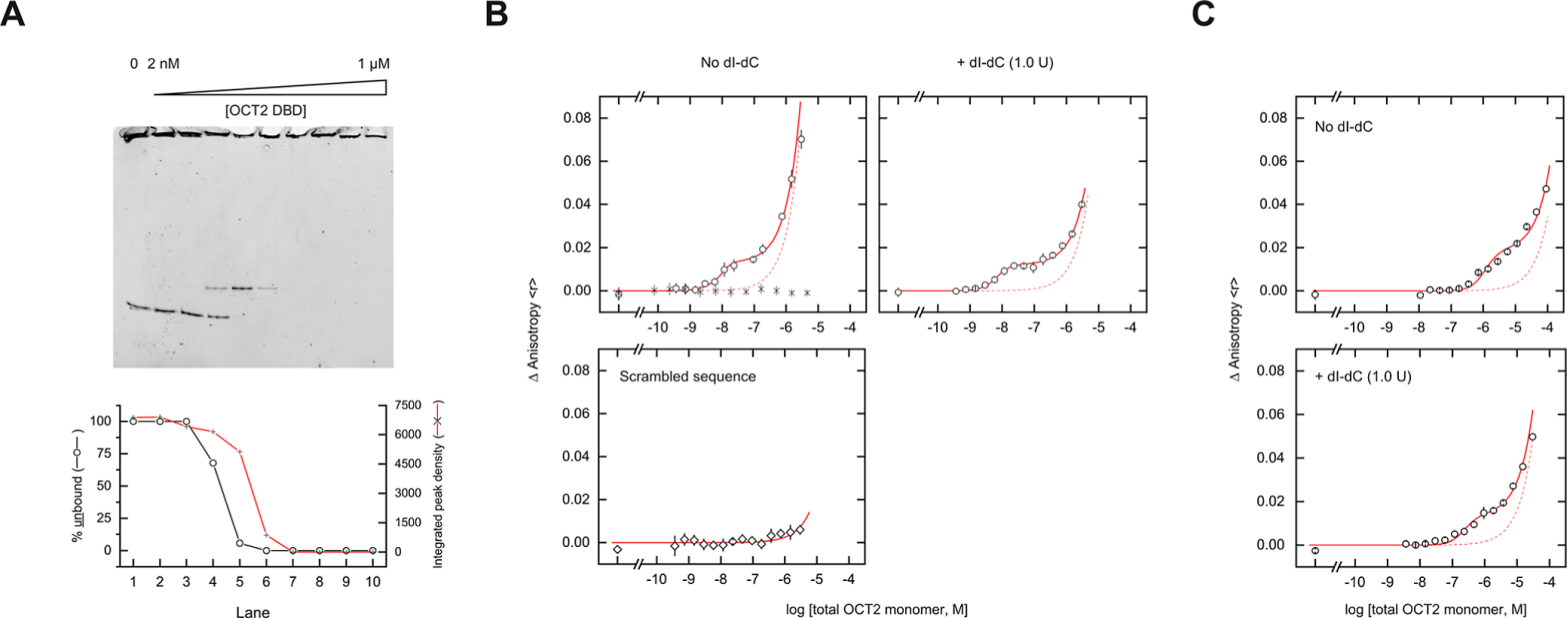
Secondary species are coupled to the site-specific OCT2/MORE complex. (A) Representative native polyacrylamide gel electrophoresis titration a 0.2 kbp DNA fragment (10 nM), harboring a single copy of the MORE site, by 2-fold dilutions of the OCT2 DBD from 1 μM. (B) Titrations of a 5′-HEX-labeled MORE (○) or scrambled-sequence probe (◇; 5′-CTACTTCCGTATACGGAAGTAG-3′) with OCT DBD at 0.15 M Na^+^ with and without 1 U poly[d(I-C)]. Symbols represent means ± SD of the baseline-subtracted anisotropy. Solid curves represent fits of the model expressed by eqs 1–3. Dashed curves represent inferred levels of excess binding according to the model, i.e., ϕ[O] in eq 1. The × symbols represent a titration under the same conditions in the presence of 10 μM unlabeled MORE. (C) Titrations with OCT DBD at 0.35 M Na^+^.

To probe the formation of the OCT2/MORE complex in solution, we titrated a 0.2 kbp DNA fragment harboring a single MORE site with the OCT2 DBD. Resolution by native polyacrylamide gel electrophoresis revealed a single discrete bound band (Figure 2A). The absence of two discrete bands showed that the singly bound state was not significantly populated at equilibrium and a highly cooperative formation of the 2:1 OCT2/DNA complex at the MORE site. With increasing OCT2 concentration, the DNA became insensitive to staining (by an ethidium homodimer) due to excess OCT2 binding.

To test whether the excess protein binding was related to the MORE site or incidental nonspecific DNA binding, we carried out fluorescence polarization titrations of a 5′-HEX-labeled version of the 22-bp MORE sequence used in the cocrystal structure. Saturable binding to the site-specific oligonucleotide was also accompanied by excess binding that was unsaturable within the range of OCT2 concentrations tested (Figure 2B). In accord with the electrophoretic mobility data, we modeled the saturable component as positively cooperative formation of an OCT2 homodimer per MORE site, as characterized by the macroscopic dissociation constant *K*_D_
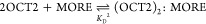
4

As detailed in the Materials and Methods, the excess anisotropy was treated as linearly dependent on free OCT2 concentration and parameterized by the empirical parameter ϕ. At 0.15 M Na^+^, OCT2 bound the MORE site with *K*_D_ = 3.2 ± 1.4 nM, and the excess anisotropy change exceeded that of the specific component by sub μM concentrations of OCT2. We observed no systematic quench in the fluorescence intensity in the titration to suggest a photophysical artifact of OCT2 interactions with the 5′-HEX label. To test whether the excess binding was nonspecific or coupled to the site-specific complex, we examined binding in the presence of a saturating concentration (10 μM) of unlabeled MORE site. We observed quantitative inhibition of binding to the probe (×symbols in Figure 2B), indicating that the excess binding was not nonspecific. Furthermore, binding to an off-target probe was negligible relative to that of the MORE sequence. The excess binding as reported by ϕ thus represented secondary complexes that were contingent on the MORE-specific binding and suggested the potential for OCT2 to cross-link MORE-bound complexes in solution as well as in crystallo.

To authenticate and probe the impact of nonspecific binding on the OCT2/MORE complex, we measured binding in the presence of 1.0 U poly[d(I-C)], an excess (∼7 mM bp) of noncognate DNA relative to the high-affinity MORE probe (5 nM). At 0.15 M Na^+^, the site-specific affinity was slightly reduced (*K*_D_ = 5.5 ± 1.3 nM), despite a 3-fold drop in secondary loading ϕ. The secondary complexes therefore did not divert OCT2 from the site-specific complex under these conditions. To probe the role of the nonspecific contributions further, we determined the *K*_D_ and secondary loading ϕ at a higher Na^+^ concentration. At 0.35 M Na^+^ (Figure 2C), *K*_D_ was highly salt-sensitive in the absence of noncognate DNA, dropping 10^3^-fold (*K*_D_ = 1.2 ± 0.3 μM), while ϕ was suppressed by 2 orders of magnitude. In sharp contrast with the low-salt condition, the presence of poly[d(I-C)] enhanced site-specific binding at 0.35 M Na^+^ by ∼10-fold (0.18 ± 0.06 μM) and increased ϕ by ∼4-fold. OCT2/MORE interactions thus exhibited divergent electrostatic properties and sensitivity to site-specific versus nonspecific DNA.

### Intersegmental Transfer of OCT2 Is Tunable by Electrostatics

**Figure 3:**
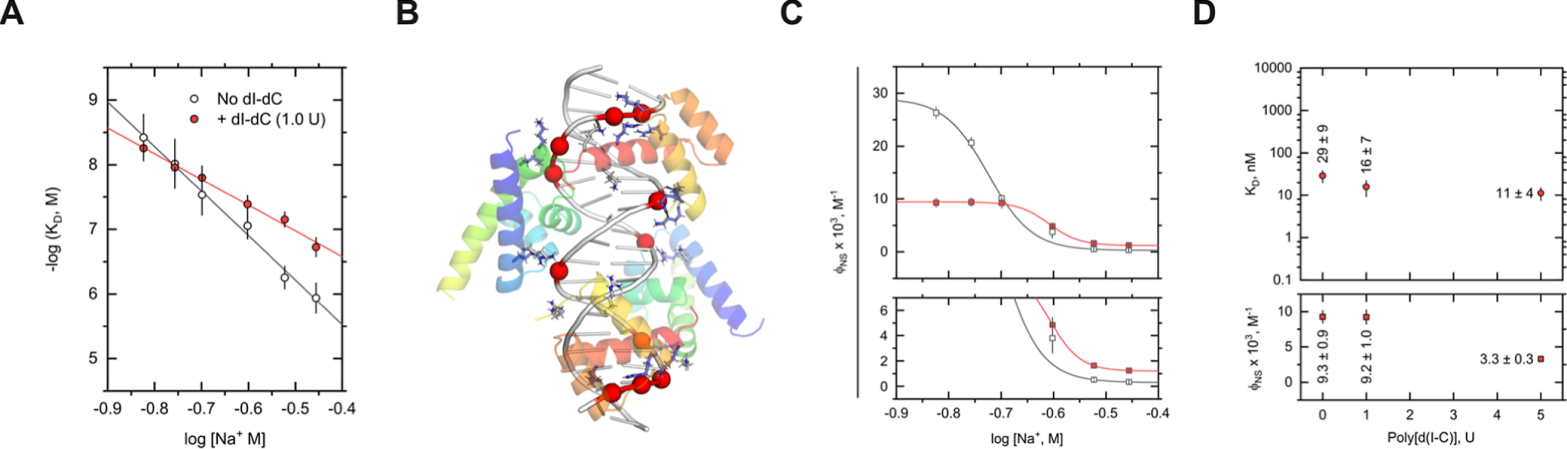
Ionic perturbations unmask coupling of nonspecific binding to the OCT2/MORE complex. (A) Salt dependence of the macroscopic equilibrium dissociation constant, *K*_D_. (B) Ionic OCT2 contacts (sticks with blue C atoms) with DNA phosphates (red spheres) within a 4 Å distance cutoff. (C) Salt dependence of secondary MORE complexes ϕ. (D) Insensitivity of *K*_D_ to poly[d(I-C)] at a Na^+^ concentration (0.2 M) where compensatory effects from poly[d(I-C)] are essentially balanced. The logarithmic ordinate of the upper *K*_D_ plot is spaced identically as Panel A.

Given the unexpected affinity enhancement in the binding of OCT2/MORE by poly[d(I-C)] at high salt, we determined the complete salt dependence of *K*_D_ and ϕ over the range of 0.15 to 0.35 M Na^+^. With or without poly[d(I-C)], *K*_D_ exhibited a linear log–log (power law) relationship as a function of Na^+^ concentration. Per linkage thermodynamics,^31^ the signs of the slopes indicated net ion release () relative to unbound OCT2 and DNA, while their magnitudes differed significantly (Figure 3A and Table 1). Contribution to the observed ion release (Δ*m*) was dissected starting with the counterion release from neutralized DNA phosphates, as provided by polyelectrolyte theory
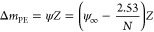
5where the parameter ψ reflects screening and condensation interactions between (signed) *Z* charges on backbone phosphates and their ion atmosphere.^32^ The assigned value of ψ = 0.75 includes an end-effect correction^33^ for the *N* = 20 (phosphate-bearing) bp in the MORE oligonucleotide relative to polymeric DNA (ψ_∞_ = 0.88). An estimate based on 11 backbone phosphates (*Z* = −11) found within 4 Å of a basic protein residue in the cocrystal structure (Figure 3B) yielded Δ*m*_PE_ = −8.3, compared with observed slope SK = −6.8 ± 0.3 (per OCT2 dimer) without poly[d(I-C)]. The remaining positive contribution, Δ*m*_other_ = SK – Δ*m*_PE_ = 1.5 ± 0.3, pointed to contributions involving only proteins from ion uptake upon binding, such as a loss of salt bridges.

**Table 1:** Salt Dependence of MORE-Specific Binding Affinity for OCT2 (K_D_)

		*K*_D_, M		log(*K*_D_, M)	
[Na^+^], M	log[Na^+^, M]	–poly[d(I-C)]	+poly[d(I-C)]	–poly[d(I-C)]	+poly[d(I-C)]
0.15	–0.82	(3.8 ± 1.4) × 10^–9^	(5.5 ± 1.3) × 10^–9^	–8.42 ± 0.35	–8.26 ± 0.10
0.175	–0.76	(9.7 ± 3.7) × 10^–9^	(11 ± 4) × 10^–9^	–8.01 ± 0.38	–7.96 ± 0.17
0.20	–0.70	(2.9 ± 0.9) × 10^–8^	(1.6 ± 0.7) × 10^–8^	–7.53 ± 0.32	–7.80 ± 0.18
0.25	–0.60	(8.8 ± 1.8) × 10^–8^	(4.1 ± 1.3) × 10^–8^	–7.05 ± 0.20	–7.39 ± 0.14
0.30	–0.52	(5.6 ± 1.0) × 10^–7^	(0.71 ± 0.19) × 10^–7^	–6.25 ± 0.17	–7.15 ± 0.12
0.35	–0.46	(12 ± 3) × 10^–7^	(1.9 ± 0.6) × 10^–7^	–5.93 ± 0.23	–6.73 ± 0.15
					
			*SK*	–6.8 ± 0.3	–4.0 ± 0.2

In the presence of 1.0 U poly[d(I-C)], SK was reduced by ∼40% to −4.0 ± 0.2. As predicted by the opposite effect of poly[d(I-C)] on *K*_D_ from 0.15 to 0.35 M Na^+^, the salt dependencies in *K*_D_ with and without poly[d(I-C)] crossed near 0.175 M Na^+^ and diverged monotonically with the Na^+^ concentration (Figure 3A). The deleterious effect of poly[d(I-C)] on *K*_D_ and ϕ below 0.175 M Na^+^ was ascribed to a depletion of unbound OCT2 to nonspecific binding sites to poly[d(I-C)]. If this were the only contribution by the nonspecific (NS) DNA, however, as has been reported for non-POU systems,^34^ one would expect *K*_D_ to converge with salt (given the vast excess of nonspecific DNA), not the divergent behavior as observed here. As MORE and poly[d(I-C)] were present as separate DNA duplexes, any effect by poly[d(I-C)] on *K*_D_ must relate to an intersegmental exchange of the OCT2 probe between the poly[d(I-C)] and the MORE probe
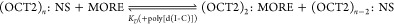
6

As the equilibrium was shifted to the right with increasing Na^+^ concentration, the improvement in *K*_D_ by poly[d(I-C)] above the crossover Na^+^ concentration indicated a favorable contribution from intersegmental transfer with opposite electrostatic properties (i.e., ion uptake) relative to direct specific binding. Thus, opposing mechanisms viz-à-viz depletion of unbound protein versus intersegmental transfer compensated to yield a linear log–log relationship with Na^+^ concentration.

To gain more evidence for this picture, we analyzed the dependence of ϕ on the Na^+^ concentration. In absolute magnitude, ϕ was markedly suppressed with increasing Na^+^ concentration but strongly increased in relative terms by poly[d(I-C)] (Figure 3C and Table 2). We tested for the presence of compensation directly by further increasing the concentration of poly[d(I-C)] by 5-fold (to 5 U) at 0.2 M Na^+^, near the crossover point for *K*_D_ and ϕ. Compared with 1 U poly[d(I-C)], site-specific binding was minimally perturbed despite a further 3-fold reduction in ϕ (Figure 3D). Compensation was essentially complete within a narrow ionic range between 0.175 and 0.2 M Na^+^.

**Table 2:** Salt Dependence of Nonspecific Loading of OCT2 at the MORE Site (ϕ)

		ϕ, M^–1^		
[Na^+^], M	log[Na^+^, M]	–poly[d(I-C)]	+poly[d(I-C)]	Δ
0.15	–0.82	(2.6 ± 0.1) × 10^4^	(0.93 ± 0.09) × 10^4^	–65%
0.175	–0.76	(2.1 ± 0.1) × 10^4^	(0.94 ± 0.08) × 10^4^	–55%
0.20	–0.70	(1.0 ± 0.1) × 10^4^	(0.92 ± 0.10) × 10^4^	–8%
0.25	–0.60	(3.8 ± 1.2) × 10^3^	(4.8 ± 0.6) × 10^3^	+28%
0.30	–0.52	(0.52 ± 0.07) × 10^3^	(1.6 ± 0.2) × 10^3^	+217%
0.35	–0.46	(0.34 ± 0.03) × 10^3^	(1.2 ± 0.1) × 10^3^	+264%

### Molecular Dynamics Simulations of OCT2/DNA Complexes

Characterization by gel mobility shift and fluorescence anisotropy showed a discrete OCT2/MORE complex accompanied by secondary binding complexes in solution. As the titrations range from concentrations at which MORE sites are in excess to concentrations at which OCT2 is in excess, the results show clearly that secondary complexes are significantly populated at subsaturating OCT2 concentrations. Since the cocrystal structure suggested cross-linking between complexes, the linkage of the POU_X_ subdomains in the discrete OCT2/MORE complex remained an open question. In addition to the axial linkage of the two subdomains, the OCT2 linker could also be modeled radially around the DNA helix, in trans or cis with respect to each other (Figure S2A). We tested these linker topologies in explicit-solvent molecular dynamics (MD) simulations. Axial linkages were more stable in potential energy than radial ones (Figure S2B) and resulted in locally distinct conformational dynamics (Figure S2C). However, the dynamics of the two radial linkage modes were similar in magnitude to axial linkage. In silico results thus indicated that the POU_S_ subdomains favored an axial linkage in the MORE-bound complex.

**Figure 4:**
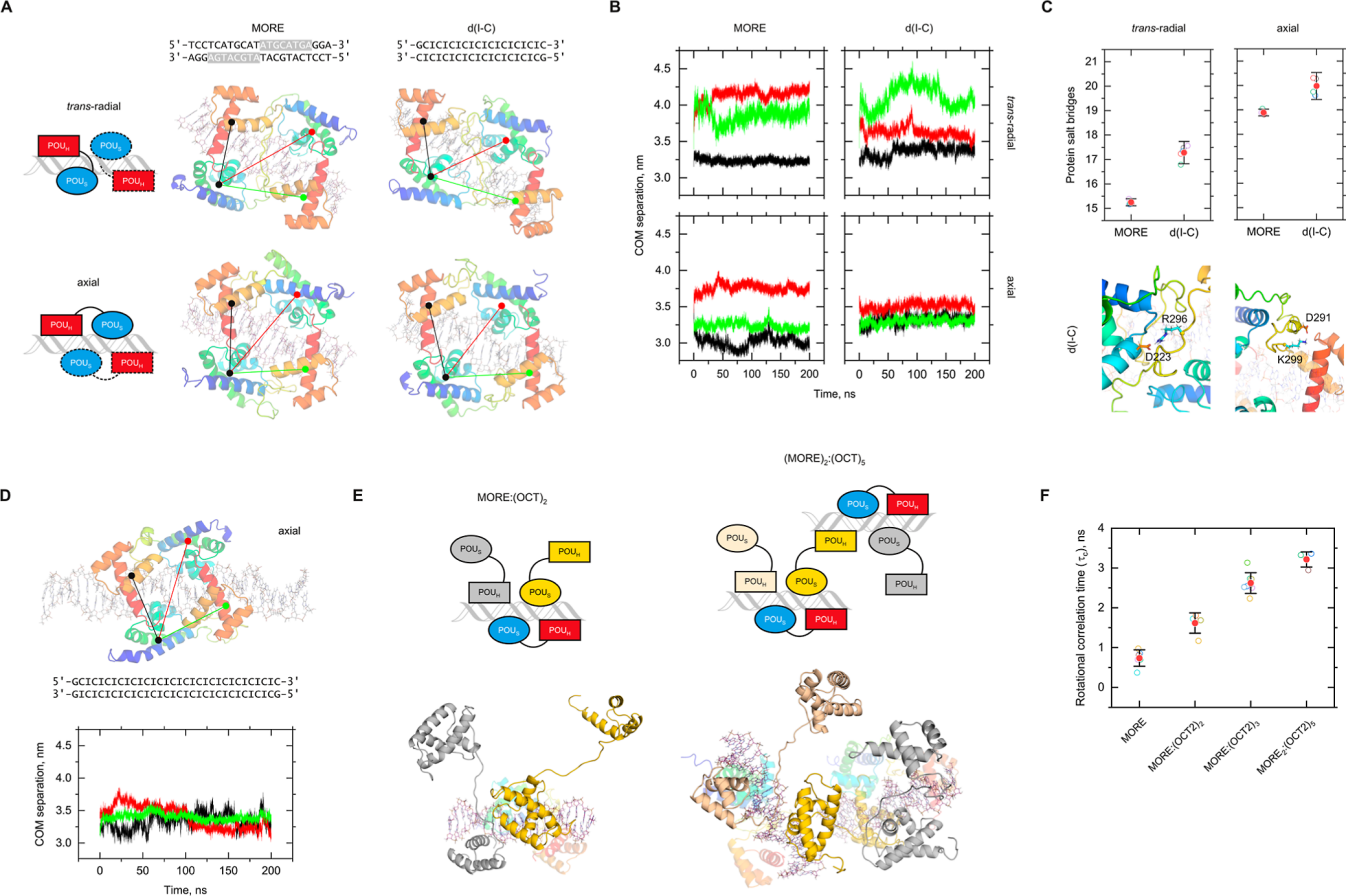
Exploring secondary OCT2/DNA complexes by molecular dynamics simulations. (A) Homology models of nonspecific OCT2/DNA complexes from the MORE-bound complex in two contrasting linker topologies. To mitigate artifactual fraying in the d(I-C) sequence, the terminal bp was replaced with GC. Colored lines show the center-of-mass (COM) separation from one POU_S_ to the other three POU_X_ subdomains. (B) Representative trajectories of COM separation over 0.2 μs following the black–red–green color scheme as used in Panel A. Note the selective closing of both d(I-C)-bound POU_S_ subdomains. (C) Mean ± SD of salt-bridge contacts (complementary N and O atoms from Asp/Glu and Arg/Lys side chains plus the N- and C-termini) within a 4 Å cutoff. Open symbols represent averages from separate simulations. (D) Dynamics of axially linked OCT2 on a d(I-C) sequence doubled in length. (E) Model of a MORE site with one fully bound axially linked OCT2 and two partially bound OCT2 monomers, as well as a supramolecular dimer. For clarity, the fully bound OCT2 subunits were rendered semitransparent. (F) Averaged rotational correlation times ±SD computed in nonoverlapping 10 ns windows using vectors defined by the terminal three P atoms in each DNA strand to approximate the HEX label in experimental probe.

Against this backdrop, the differential salt dependence of nonspecific binding of the NMR spectroscopy of the OCT2/DNA suggests structural differences from the site-specific complex. While no structure of a nonspecific POU/DNA complex has been reported to our knowledge, NMR residual dipolar coupling measurements suggest that the POU_S_ subdomain is conformationally altered on nonspecific oligomeric DNA.^20^ We carried out explicit-solvent MD simulations on a homology model of the OCT2/DNA cocrystal structure with the MORE sequence substituted with alternating d(I-C) to simulate poly[d(I-C)]. To capture potential topologic heterogeneity in nonspecific binding, we examined two arrangements in which the linkers were axial and trans-radial (the latter exhibiting both radial topologies) (Figure 4A).

Over 0.2 μs of simulation (Figure 4B), the POU_X_ subdomains in all the complexes maintained DNA binding with negligible change in the tertiary structure. However, there were significant shifts in their topology as the two POU_S_ subdomains in the nonspecific complexes moved toward each other relative to their MORE-bound counterparts: by ∼7 Å (center-of-mass separation) in the radially linked homodimer and ∼3 Å in the axially linked homodimer. As a result, the nonspecifically bound OCT2 gained salt bridges over MORE-bound OCT2 in both linkages (Figure 4C). These fluctuating salt bridges included new solvated contacts by a cluster of basic residues (RRRKKR; residues 296 to 301), near the C-terminal end of the OCT2 linker (Figure 1A), with acidic residues that were brought into proximity by the approaching POU_S_ subdomains. A loss of these salt bridges in a transition of these nonspecific complexes to the MORE-bound complex would be associated with ion uptake, in accordance with the experimental effect of poly[d(I-C)] on *K*_D_. To test if the selective shifts of the POU_S_ subdomains were related to the length of the DNA, we simulated a nonspecific complex docked on d(I-C) that was double in length and observed similar results (Figure 4D).

We turned next to the secondary MORE-bound species that were responsible for the excess fluorescence anisotropy change in the titrations. Motivated by the in crystallo data, we envisioned the secondary species as an oversaturated ensemble of cross-linked OCT2/MORE complexes and partially unbound OCT2. Given the propensity of POU domains for intersegmental transfer,^18−20^ POU_X_ subdomains dangling from partially bound OCT2 can bridge other MORE sites which POU_X_ subdomains dynamically bind and unbind. The reduced rotational diffusion of such supramolecular complexes would account for the excess fluorescence anisotropy change as steady-state anisotropy reports a population-weighted average of the thermodynamic ensemble.

To explore this notion further, we generated a realization of MORE with one fully bound OCT2 (axially linked) and two partially bound monomers as well as a supramolecular dimer with two MORE sites and five OCT2 (Figure 4E). We simulated these models and compared their rotational correlation times (τ_c_) with those of unbound MORE and the canonical OCT2/MORE complex, using the plane defined by the P atoms in the final three nucleotides of each strand as a proxy for the overall rotational contribution to the HEX label in the experimental probe. Relative to MORE DNA alone, rotational diffusion slowed with successive equivalents of the OCT2 and the OCT2/DNA complex, with τ_c_ of the supramolecular dimer more than 2-fold higher than the discrete OCT2/MORE complex (Figure 4F). As partial binding affords up to four dangling subdomains per MORE site, and fully bound OCT2 may present in axial or radial linkage, the potential for further and heterogeneous supramolecular assembly of a cross-linked network with increasing OCT2 concentrations is therefore considerable.

## Discussion

### Site-Specific OCT2 Binding Is Coupled to Secondary Complexes

In solution, site-specific binding to an optimal site such as MORE by the oxidized oxygen atom (OCT2) is coupled to secondary species at thermodynamic equilibrium. In addition to the primary homodimer/MORE complex, two other binding modes of OCT2 are identified at equilibrium. One mode consists of secondary MORE-bound complexes harboring more than two copies of the OCT2 subunits. Although this binding mode is analyzed empirically in the titration data (ϕ) as additive to the site-specific component according to eq 1, its appearance is strictly contingent on the site-specific complex (Figure 2B). Moreover, the reciprocal perturbations on ϕ and *K*_D_ by salt or poly[d(I-C)] are positively cocorrelated. Based on these experimental observations, this secondary binding mode is most consistent with oversaturated MORE-bound complexes bearing one or more partially bound OCT2 monomers
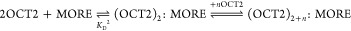
7

The entropic cost of loading additional equivalents of OCT2 in a supersaturated complex is furnished by excess concentrations of protein. While MD simulations show that these secondary complexes can include cross-linked MORE sites, their place may be substituted by other cognate (octamer) sites in the context of chromatin. In addition, dangling subdomains in partially bound OCT2 would be poised to occupy nearby octamer sites. The kinetic advantage over recruitment of an unbound monomer from bulk solution thus provides a molecular rationale for the positive correlation between ϕ and *K*_D_.

In addition to site-specific binding, OCT2 interacts avidly with poly[d(I-C)], a homogeneous nonspecific (NS) polymer. Because poly[d(I-C)] is noncontiguous with respect to the MORE probe, nonspecifically bound OCT2 cannot diffuse (slide or hop) to or from the target site but only through intersegmental transfer. Consistency with the observed positive impact of poly[d(I-C)] on *K*_D_ with increasing Na^+^ concentration requires that this transfer be thermodynamically associated with an overall ion uptake as shown in eq 6
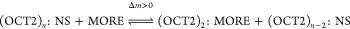


MD simulations show that ion uptake can be rationalized by a higher density of salt bridges in nonspecifically bound OCT2 relative to MORE-bound protein (Figure 4C). The selective conformational shifts in POU_S_ that lead to these excess salt bridges in the nonspecific complex are in accord with reported RDC measurements by NMR.^20^ The magnitude of molar free energies associated with solvated salt bridges in protein^35^ may readily exceed that from DNA counterion release^36^ at the Na^+^ concentrations used: Δ*G*_PE_ = -ψ*RT*ln[Na^+^] = 2 kJ/(mol ion) at 0.35 M Na^+^.

**Figure 5:**
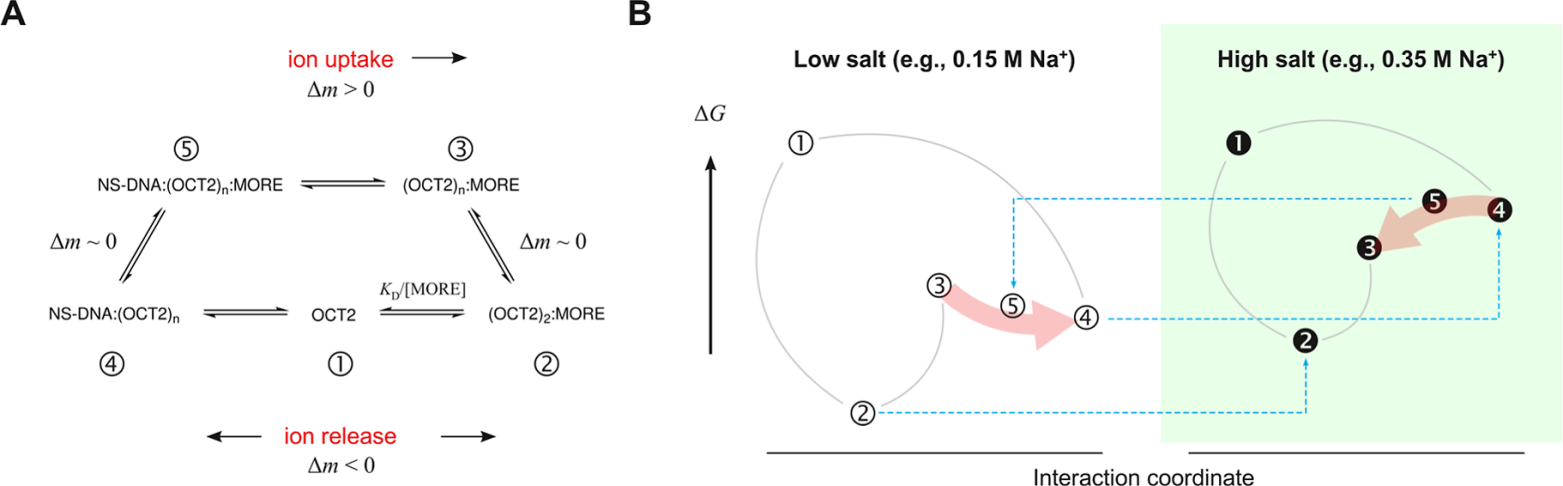
Model of thermodynamic coupling to the OCT2/MORE complex. (A) Five major macroscopic species accounts for the observed equilibrium perturbations of site-specific affinity (*K*_D_) and secondary loading (ϕ) by salt and the nonspecific DNA polymer poly[d(I-C)]. See text for rationale for assignment of ion deposition. (B) Use of the proposed model to interpret the experimental equilibrium titration data. Curves connect the equilibrium species introduced in Panel A. Blue dashed arrows denote the relative experimental and inferred salt-dependent shifts in free energy of the MORE- and nonspecifically bound complex. The red arrows highlight the reversal by salt of the relationship between the secondary and nonspecific OCT2, which differ in electrostatic properties. The placement of the species on the free energy scale is intended only to indicate the rank order of the species of interest under the two salt conditions as prompted by their experimentally observed changes.

To organize the experimental inferences expressed in eqs 6 and 7, we propose the following equilibrium model, schematized in Figure 5A, to describe the interactions of OCT2 with MORE and nonspecific DNA. Our rationale and assumptions are as follows:

- there are five major macroscopically populated OCT2 species in dynamic equilibrium, labeled **1** through **5**. Microstates with partially unbound OCT2 (one POU_X_ subdomain disengaged) but the same indicated protein/site stoichiometry (e.g., **3** and **4**) are treated here as indistinguishable. Free DNA is not shown explicitly for simplicity.
- Transitions from unbound protein (**1**) to DNA-bound states (**2** and **4**) are associated with ion release.
- Transition **4** ⇌ **5** relates nonspecifically bound OCT2 and a MORE probe with one or more complementary subsite(s). We assume that the intersegmentally cross-linked species **5** is an equilibrium intermediate for the purpose of the present discussion. As both nonspecific DNA and MORE are involved, with no net change in OCT2 occupancy, we take Δ*m* ∼ 0.
- Similarly, the secondary MORE-bound species (**3**) is saturated with respect to DNA. Since the transition **3** ⇌ **2** does not alter the net saturation of the MORE site, we treat Δ*m* ∼ 0.
- Based on the previous two features, the transition **5** ⇌ **3** is associated with ion uptake (Δ*m* > 0) in the direction of the secondary MORE-bound species (**3**), to be consistent with eq 6.

Armed with the proposed scheme, we proceeded to interpret the salt-dependent titration data in better detail (Figure 5B). Under low salt conditions in which poly[d(I-C)] impairs MORE-specific binding, the secondary MORE-bound species **3** is less favorable than the cross-linked intermediate **5** as the former requires ion uptake. Since **5** is also less favorable than the nonspecific complex **4** due to the translational entropic cost of cross-linking two DNA strands, we expect nonspecific binding to deplete the secondary species **3**, in accord with the observed effect of poly[d(I-C)] on ϕ, in addition to depleting unbound OCT2 for MORE binding.

To explain the improvement in *K*_D_ by poly[d(I-C)] under high-salt conditions, we invoke the nonspecific complex, which harbors more ionic contacts and is therefore more salt sensitive. We therefore interpret the crossover in *K*_D_ and ϕ (observed at ∼0.175 M Na^+^) as preferential destabilization of nonspecific OCT2 (**4**) that inverts the energetic relationship between **5** and **3**, setting up intersegmental transfer from poly[d(I-C)] (via **3**) as a DNA-facilitated pool of OCT2 toward the target MORE-specific complex **2** (Figure 5B). The increased contribution from DNA-facilitated translocation thus accounts for the more favorable *K*_D_ in the presence of poly[d(I-C)].

## Conclusions

In-solution characterization of purified OCT2 DBD, an optimal DNA site (MORE) and homogeneous poly(d(I-C)) polymer, revealed secondary complexes that couple to the site-specific dimeric complex in accord with structural heterogeneity in the OCT2/MORE cocrystal structure. These secondary species are contingent on site-specific binding and represent the loading of additional equivalents of protein and cross-linked dimeric OCT2/DNA complexes. To organize the inferences drawn from experimental data and MD simulations, we proposed an equilibrium model that provides a framework for understanding the relationship among site-specific, secondary, and nonspecific DNA binding. Given the well-conserved bipartite POU architecture, this model for OCT2 should be generalizable to other POU members. However, the extended length of the OCT2 linker may facilitate the coupled interactions in a greater range and combinatorial possibilities over POU factors harboring shorter linkers or encoding structure-forming elements.

The identification of secondary specific binding of the POU dimer extends reported MD^18^ and NMR^19,20^ characterization of the monomer in intersegmental transfer to and from a single DNA octamer motif. Dimeric binding is a major element in the POU/DNA repertoire,^22^ and the present work suggests enhanced propensity for dimeric POU domains to cross-link DNA over their monomeric counterpart. In NMR experiments,^20^ where analytes interacting monomerically are present at essentially equimolar 10^–5^ M concentrations, cross-linked DNA constitutes sparsely populated states at less than 1% abundance. Here, the prominence of secondary species over much more dilute concentrations relevant to dimeric MORE-specific binding, under all solution conditions examined, attests to their physical significance in POU/DNA recognition and offers a basis for the complex patterns observed in genomic POU-binding motifs.^37,38^

## Supporting Information Available

Collection and refinement statistics of the OCT2/MORE co-crystal structure, unweighted residual sum of squares in fits to the fluorescence polarization data, the recombinant DNA fragment used in gel mobility shift experiments, and MD simulations of OCT2/MORE complexes in alternative subdomain linkages (PDF)
