# Supplementary material for: Coupled Heterogeneity to Dimeric Site-Specific Binding by the POU-Family Transcription Factor OCT2
Source: J Phys Chem B. 2025 Feb 17;129(8):2138–48. doi: 10.1021/acs.jpcb.4c07071 (PMC11873960; doi:10.1021/acs.jpcb.4c07071)
Supplement: Supplementary file 1 — jp4c07071_si_001.pdf [file jp4c07071_si_001.pdf]

SUPPORTING INFORMATION

Coupled Heterogeneity to Dimeric Site-Specific Binding by the POU-family Transcription Factor OCT2

January 23, 2025

J. Ross Terrell † and Gregory M. K. Poon *

Department of Chemistry

Georgia State University

Atlanta, GA 30303, USA

† Present address: U.S. Army Medical Research Institute of Chemical Defense, 8350 Ricketts Point Road, Aberdeen Proving Ground, MD 21010-5400

* To whom correspondence should be addressed at: P.O. Box 3965, Atlanta, GA 30302-3965, USA. Email: gpoon@gsu.edu.

INDEX TO SUPPORTING INFORMATION

Table S1. Collection and refinement statistics of the OCT2/MORE co-crystal structure S3
Table S2. Unweighted residual sum of squares (RSS) in fits to the fluorescence polarization data S4
Figure S1. Recombinant DNA fragment used in the gel mobility shift experiments S5
Figure S2. MD simulations of OCT2/MORE complexes in alternative subdomain linkages S6

Table S1. Collection and refinement statistics of the OCT2/MORE co-crystal structure. All distance-related measures are based on Å.

**PDB ID**	9DZM
**Wavelength**	0.9201
**Resolution range**	33.91 - 2.54 (2.631 - 2.54)
**Space group**	P 1
**Unit cell**	38.029 54.922 69.164 82.373 79.657 71.769
**Total reflections**	33917 (3233)
**Unique reflections**	16395 (1603)
**Multiplicity**	2.1 (2.0)
**Completeness (%)**	95.45 (95.19)
**Mean I/sigma(I)**	8.37 (2.03)
**Wilson B-factor**	51.55
**R-merge**	0.06205 (0.4024)
**R-meas**	0.08414 (0.544)
**R-pim**	0.0564 (0.3637)
**CC1/2**	0.995 (0.87)
**CC***	0.999 (0.965)
**Reflections used in refinement**	16396 (1603)
**Reflections used for R-free**	1640 (160)
**R-work**	0.2172 (0.3602)
**R-free**	0.2418 (0.3745)
**CC(work)**	0.946 (0.868)
**CC(free)**	0.940 (0.866)
**Number of non-hydrogen atoms**	3127
**macromolecules**	3092
**ligands**	1
**solvent**	34
**Protein residues**	275
**RMS(bonds)**	0.005
**RMS(angles)**	0.89
**Ramachandran favored (%)**	95.88
**Ramachandran allowed (%)**	2.25
**Ramachandran outliers (%)**	1.87
**Rotamer outliers (%)**	2.51
**Clashscore**	5.01
**Average B-factor**	66.93
**macromolecules**	70.92
**ligands**	138.54
**solvent**	43.92

Table S2. Unweighted residual sum of squares (RSS) in fits to the fluorescence polarization data. Fluorescence anisotropy data is the mean of three or more experiments. The fitting model consists of partitioning of the measured anisotropy into saturable and unsaturable components, Eq. (1); the Hill equation for the saturable component, Eq. (2); and depletion of the probe, Eq. (3).

		Unweighted RSS		
$[Na^+]$, M	log $[Na^+$, M]	– poly[d(I-C)]	+ 1.0 U poly[d(I-C)]	+10 U poly[d(I-C)]
0.15	-0.82	1.36651×10^{-5}	1.10208×10^{-5}	
0.175	-0.76	1.24593×10^{-5}	4.36508×10^{-5}	
0.20	-0.70	1.86460×10^{-5}	7.49658×10^{-6}	7.48509×10^{-6}
0.25	-0.60	1.23533×10^{-5}	8.14178×10^{-6}	
0.30	-0.52	1.18443×10^{-5}	1.18443×10^{-5}	
0.35	-0.46	5.85987×10^{-5}	2.49617×10^{-5}	

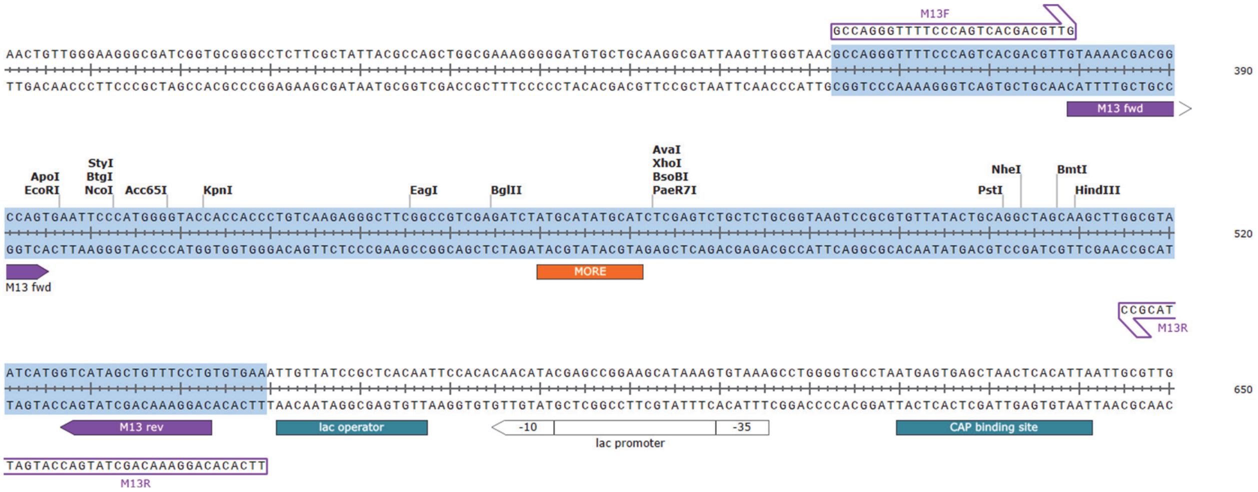

Figure S1. Recombinant DNA fragment used in the gel mobility shift experiments. An insert harboring the MORE sequence was cloned into the EcoRI and HindIII sites of pUC19. PCR amplification with the indicated M13 primers yields the 0.2 kbp fragment (highlighted) used as DNA probe. Shown restriction sites are unique in the plasmid.

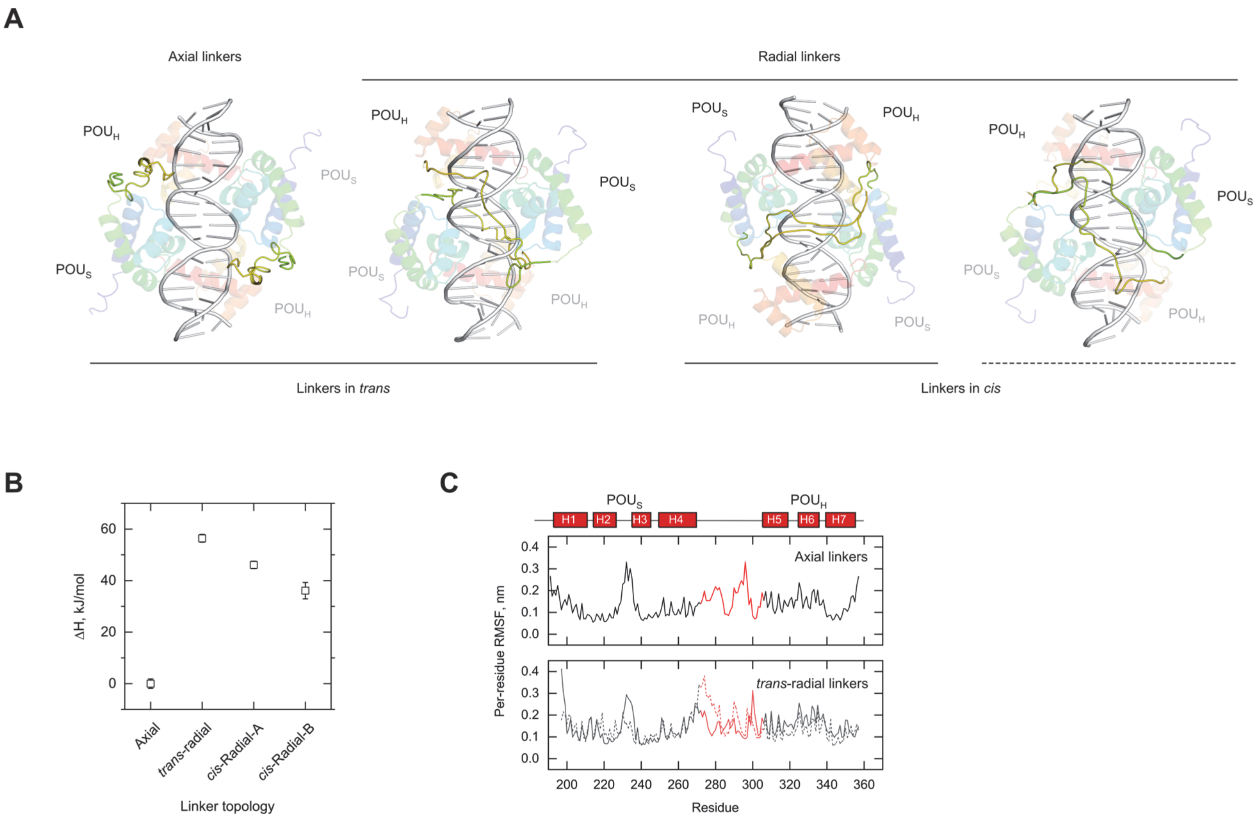

Figure S2. MD simulations of OCT2/MORE complexes in alternative subdomain linkages. **A)** Linker-inserted homology models of the OCT2/MORE complex using the co-crystal structure as template. Non-linker portions of the proteins are rendered semi-transparent to aid visualization. **B)** Difference in enthalpy (potential energy) of compositionally identical *NPT* ensembles consisting of one copy of the OCT2/MORE complex, 21,222 TIP3P water molecules, 93 Na^+ and 65 Cl^- ions. **C)** Per-residue RMS fluctuations of axially (averaged) and trans-radially linked OCT2 in the MORE-bound complex. The two radial modes (solid and dashed lines) were plotted according to the scheme shown in Panel A.
